# Application of Terrestrial Laser Scanning (TLS) in the Architecture, Engineering and Construction (AEC) Industry

**DOI:** 10.3390/s22010265

**Published:** 2021-12-30

**Authors:** Chao Wu, Yongbo Yuan, Yang Tang, Boquan Tian

**Affiliations:** Faculty of Infrastructure Engineering, Dalian University of Technology, Dalian 116024, China; wuchao626@mail.dlut.edu.cn (C.W.); tangyang@mail.dlut.edu.cn (Y.T.); tian_boquan@mail.dlut.edu.cn (B.T.)

**Keywords:** terrestrial laser scanning (TLS), point cloud, citespace, architecture, engineering and construction (AEC)

## Abstract

As a revolutionary technology, terrestrial laser scanning (TLS) is attracting increasing interest in the fields of architecture, engineering and construction (AEC), with outstanding advantages, such as highly automated, non-contact operation and efficient large-scale sampling capability. TLS has extended a new approach to capturing extremely comprehensive data of the construction environment, providing detailed information for further analysis. This paper presents a systematic review based on scientometric and qualitative analysis to summarize the progress and the current status of the topic and to point out promising research efforts. To begin with, a brief understanding of TLS is provided. Following the selection of relevant papers through a literature search, a scientometric analysis of papers is carried out. Then, major applications are categorized and presented, including (1) 3D model reconstruction, (2) object recognition, (3) deformation measurement, (4) quality assessment, and (5) progress tracking. For widespread adoption and effective use of TLS, essential problems impacting working effects in application are summarized as follows: workflow, data quality, scan planning, and data processing. Finally, future research directions are suggested, including: (1) cost control of hardware and software, (2) improvement of data processing capability, (3) automatic scan planning, (4) integration of digital technologies, (5) adoption of artificial intelligence.

## 1. Introduction

In the context of “Industry 4.0”, the architecture, engineering and construction (AEC) industry is undergoing a significant shift from conventional labor-intensive practices towards digitalization and intelligence [1]. The rapid development and application of information technologies is bringing about unprecedented changes in the AEC industry. Especially in recent years, with hot discussions about the concept of smart construction and the technologies of artificial intelligence (AI) and digital twins (DT) [2,3,4,5], various information technologies have been introduced in the AEC industry to improve the productivity and management efficiency of construction, including BIM [6], RFID [7], photogrammetry [8], Internet of Things [9], cloud computing [10], blockchain [11], etc.

For the last three decades, TLS has been used incrementally with success in the AEC industry, with continuous development in the performance of laser scanners. Especially in the last ten years, plenty of studies have been conducted to verify the potential application of TLS, which has been proven to be a promising technique. In this case, some review articles about this topic have been published. Tang et al. [12] surveyed automatic reconstruction techniques for as-built building information models from scanning point clouds and discussed their potential application to automated BIM creation. Mukupa et al. [13] reviewed the application of TLS in the monitoring of structures, including change detection and deformation monitoring. Xu et al. [14] summarized various existing methods of building reconstruction from point clouds, with a particular focus on the discussion of data acquisition and processing techniques. Dong et al. [15] provided a comprehensive review of point cloud registration methods and a large-scale benchmark dataset to support the development of cutting-edge point cloud registration methods. Wang et al. [16] compared different approaches to point cloud data acquisition and discussed the different methods for each processing procedure, including (1) data cleansing, (2) data registration, (3) data segmentation, and (4) object recognition. Xu et al. [17] reviewed the methods and applications of voxel-based point cloud representations and analyzed the potential of using voxel-based representations in the construction industry. Rashidi et al. [18] explored the applications of TLS in bridge engineering. Wang et al. [19] presented the applications of point cloud data obtained from laser scans, images, and videos in the construction industry and discussed the acquisition and processing of point cloud data. However, the current reviews have various limitations, including (1) being out of date, (2) presenting an incomplete discussion that focuses on one of the specific applications, (3) provision of insufficient analysis, with an emphasis on point cloud data processing.

The number of relevant publications has increased sharply since 2017. In consequence, this paper provides a state-of-the-art review on the application of TLS in the AEC industry. The main objectives of this review are to: (1) introduce TLS and summarize the potential benefits of TLS in the AEC industry (Section 2); (2) collect relevant papers according to a preset literature search strategy and perform scientometric analysis to reflect the trends, top journals, co-occurrence keywords, and co-citation documents of publications (Section 3); (3) generalize the current TLS-related applications in the AEC industry (Section 4); (4) analyze critical issues related to application (Section 5); (5) point out future research directions (Section 6).

## 2. TLS Technology

The first working laser with three energy level transitions was created by Theodore Maiman in 1960 [20]. However, it was not until the 1990s that the first commercial laser scanners appeared on the market. Subsequently, driven by technical advances in optics, sensors, electronics and computers, significant improvements in the performance of scanners, such as with respect to speed, accuracy, and weight, were observed over the following three decades. Especially in the last ten years, the new generation of terrestrial laser scanners has provided better performance and improved system performance [21]. For example, laser scanning technology has been integrated into total stations [22,23]. Nowadays, TLS has gradually matured through continuous improvements, and has a wide range of applications.

### 2.1. Working Principle of a Terrestrial Laser Scanner

**Figure 1:**
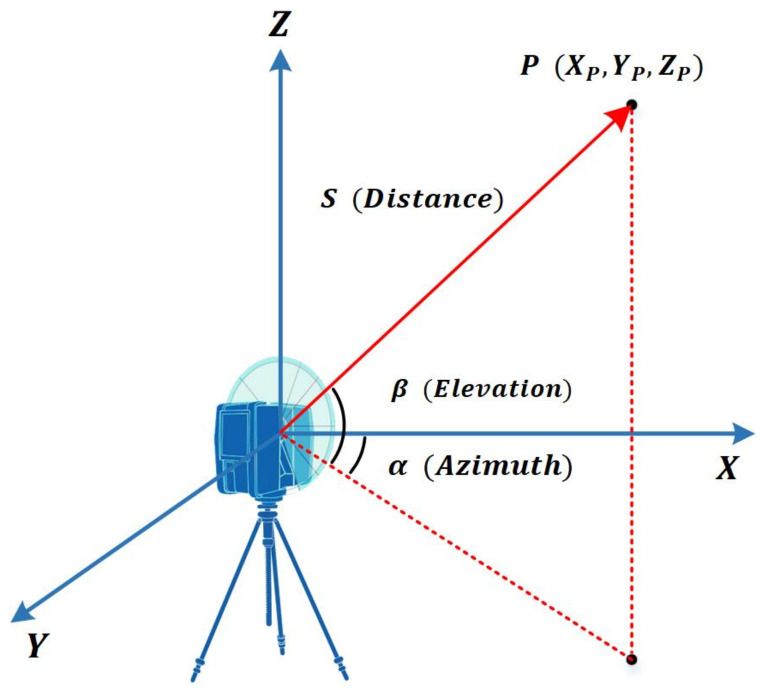
Working principle of a laser scanner.

The ranging system is the core component of a terrestrial laser scanner, and uses a laser ranger to measure the distance from the scanner to an object. The working principle is that the scanner emits a laser beam to the preset scanned area by changing the deflection angle in vertical and horizontal directions. As the laser beam hits a reflective surface in its path, it returns to the receiver. By using different methods in range measurement, the distance (*S*) between the scanner and the object can be calculated. Finally, according to the azimuthal (horizontal) and elevation (vertical) angles $\left(\alpha ,\beta \right)$ of the light, the reflecting point position $\left(Xp,Yp,Zp\right)$ can be determined by Formula (1) based on the instrument coordinate system (Figure 1).

(1) $$\left\{\begin{array}{l} X_{p}=S\cos\beta \cos\alpha \\ Y_{p}=S\cos\beta \sin\alpha \\ Z_{p}=S\cos\beta \end{array}\right.$$

Currently, there are two primary distance measurement methods used in commercial laser scanners: time-of-flight (also known as “pulse-based”) and phase-based. Each type of scanner has suitable applications in different scenarios depending on the project requirements. The strength of time-of-flight scanners lies in their much longer effective measurement range and reduced spurious point noise, while pulse-based scanners have a substantial advantage with respect to measurement accuracy and speed of acquisition. Table 1 compares the technical performance of typical scanners regarding maximum range, scan speed, and ranging accuracy.

(1) Time-of-flight: This principle is based on the classic method of recording the traveling time of a pulse of laser energy [24]. Since the speed of laser is known very precisely, if the round-trip time of the emitted pulse is recorded, the distance can be calculated using Formula (2), where c stands for the speed of laser and t the round-trip time of the light pulse.

(2) $$S=\frac{1}{2}ct$$

(2) Phase-based: The laser emits a continuous wave beam with different harmonic wavelengths typically realized by amplitude modulation (AM). The distance between a scanner and an object is determined by the shift in phase between the sent and received wave [22,25], given by Formula (3) [24]. f represents the frequency of the wave, λ is the wavelength, Δφ refers to the phase shift, N is the multiple number of full wavelengths.

(3) $$S=\frac{1}{2}\left(N\cdot \lambda +\lambda \frac{\Delta \varphi }{2\pi }\right)\;with\;\lambda =\frac{c}{f}$$

### 2.2. Potential Benefits of Using TLS in the AEC Industry

The main advantages of TLS over traditional measurement techniques include five aspects:

(1) Fast and massive sampling capability: A significant advantage of TLS is that it enables fast collection of high-density points, thus increasing productivity. Traditional instruments such as levels and total stations rely on the measurement of a limited number of points on the surface of the object [25]. Since TLS is capable of acquiring large amounts of data with speed and accuracy, it can obtain the complete surface of the object.

(2) Much more information: Apart from 3D positional information, the obtained data consist of reflected intensity values and RGB colors. With the help of this information, it is easy for an engineer to create an accurate geometric model and extract the required data, such as dimensions, spatial positioning, and structural characterization [26].

(3) Highly automated: To carry out the survey properly and effectively, it is an essential requirement for operators that they should be familiar with surveying instruments. The laser scanner is easy to use due to the high degree of automation. Learning to operate the scanner takes little time, and barely requires technical qualification.

(4) Non-contact: Since the laser beam can be reflected by most objects, the scanning process is generally non-contact. This feature contributes to improving safety in the case of hazardous environments [27] and reducing their impact on the construction process [28].

(5) Relatively high accuracy: Although the single point accuracy of TLS is usually lower than traditional techniques, it can be improved through adjustment techniques [29,30]. In addition, it can take advantage of large amounts of data to achieve better modeling accuracy [31,32].

## 3. Method

### 3.1. Literature Search and Dataset Construction

**Figure 2:**
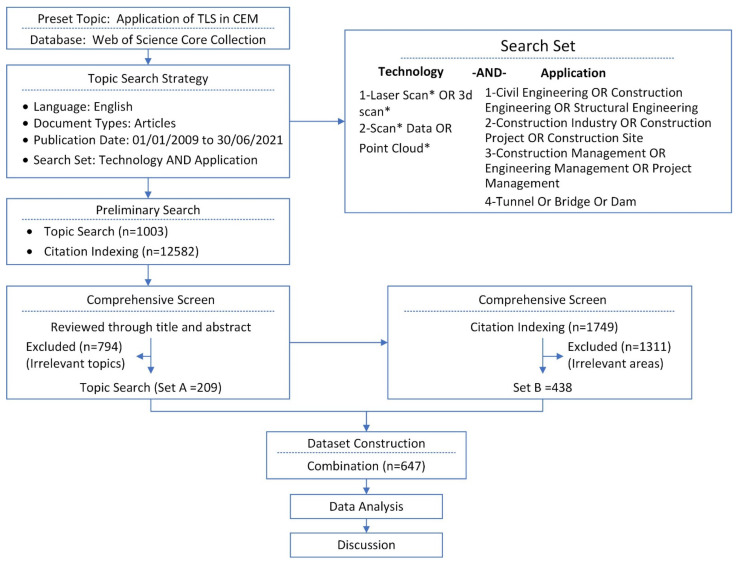
Flowchart of literature retrieval.

To ensure the adequate and accurate coverage of the research topic, the process of collecting data is performed according to the following five steps (Figure 2).

Step 1: Specifying the search scope.

To acquire the detailed records and cited references of influential articles for further analysis, the topic search is limited to original academic articles in English indexed by the Web of Science Core Collection. Only peer-reviewed journal articles are selected for analysis, because they display more rigorous and valuable contents. The topic search may miss relevant literature if the query terms are not included in the titles, abstracts, and keywords. Thus, the dataset is constructed through topic search and citation indexing to cover a more comprehensive context of the field [33]. If an article cites any of retrieved records from a topic search, the article can reasonably be considered to be thematically relevant to the research [34].

Step 2: Developing the search strategy.

The following two additional criteria are adopted to refine the search results:

(1) The publication time is from 1 January 2009 to 30 June 2021. The primary reasons for this are as follows: (1) Prior to 2009, most commercial laser scanning instruments used outdated technologies, and the application scope of TLS in the AEC field was quite restricted [21], and thus there were few meaningful papers, as demonstrated in the existing article [19]. (2) Relevant research was mainly published in conference papers with insufficient influence.

(2) The topic search is carried out by adopting the search set: (“laser scan*” OR “3D scan*” OR “scan* data” OR “point cloud*”) AND (“civil engineering” OR “construction engineering” OR “structural engineering” OR “construction industry” OR “construction management” OR “construction project” OR “construction site” Or “project management” OR tunnel OR bridge OR dam), in which the keywords were selected from the previous relevant literature. The search set is a combination of two aspects of keywords with “AND” Boolean operator: one is about laser scanning technology along with its generated data, and the other is about application fields. The wildcard asterisk (*) represents any number of characters, and is used to briefly express a word family.

Step 3: Performing a preliminary search.

A comprehensive search is initiated in accordance with the above strategy. As a result, a total of 13,585 candidate records are retrieved, including 1003 publications resulting from a topic search, and 12,582 cited articles from the creation of citation reports.

Step 4: Screening articles.

The preset topic is the application of TLS in the AEC industry. During the initial review process, any articles irrelevant to the pre-defined topic are eventually excluded.

To select data quickly, a two-stage selection plan is applied. Specifically, in the first stage, 1003 topic search records are screened by title and abstract. This step produces 209 records as Set A, which will be the main basis for conducting an in-depth discussion. In the second stage, the search is expanded by citation indexing, and excludes the papers irrelevant to the subject area. This step generates 438 records as Set B, which is adopted to perform the scientometric analysis in combination with Set A.

Step 5: Constructing the dataset.

Finally, the combined dataset is reduced to 647 publications. Each of the output records contains complete data for subsequent analysis. For example, authors, title, source, keywords, cited references, etc.

### 3.2. Analysis of Publications

To select the journals that have contributed the most to studies on the topic, a publication analysis was carried out on the basis of Set A.

**Figure 3:**
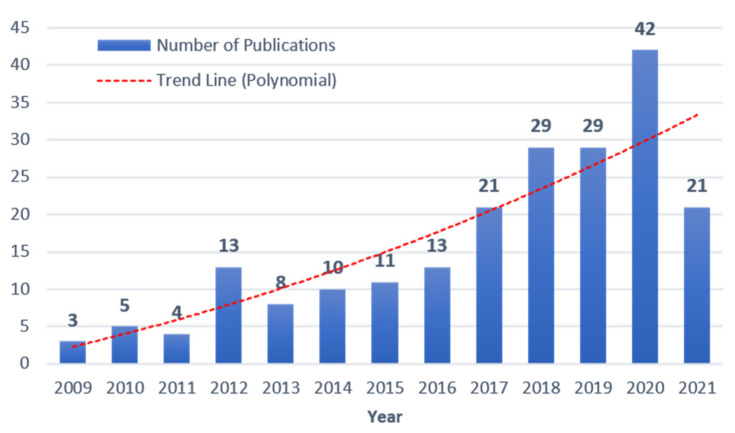
Annual publications from 2009 to 2021 (half-year).

**Figure 4:**
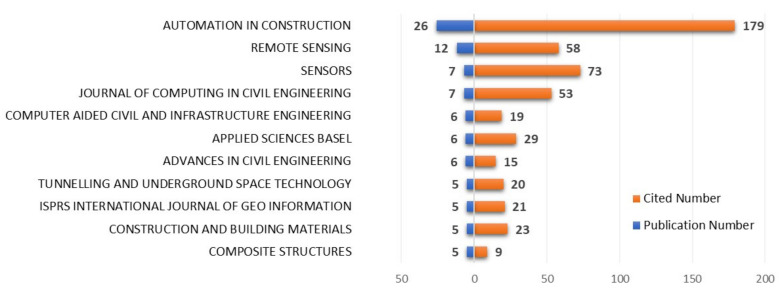
Top 10 journals in terms of publication number and number of citations.

(1) Figure 3 reveals the trend of the annual relevant publications. Although there are fluctuations every year, the number of publications showed an upward trend during the period 2009–2021, which indicates that the application of terrestrial laser scanning is attracting increasing attention from researchers in the AEC industry. The number of influential publications has increased rapidly since 2009, benefitting from improvements in the technical performance of commercial scanners. Particularly starting from 2017, the quantities increase, accounting for approximately 64.4% of the total published papers (excluding 2021). The publications in the first half of 2021 surpassed the annual publications before 2016. These data indicate that the topic is attracting increasing attention, with the expectation that informatization technologies will be introduced into the AEC industry.

(2) Figure 4 shows the top journals with the highest number of relevant papers and the number of articles cited by citation indexing in these journals. These journals have made great contributions to the development of the related research. As can be seen, the top three journals (including those tied for place no. 3), accounting for 24.9% of publications in the dataset, are *Automation in Construction*, *Remote Sensing*, *Sensors* and *Journal of Computing in Civil Engineering*. All of these journals have a five-year impact score larger than 3.0, which fully reflects the continuity and stability of the journal’s influence. Moreover, the articles from these journals are more inclined to be cited by scholars in related fields.

Traditional review methods strongly rely on domain experts’ individual decisions, which leads to the results being subjective [35,36]. In this paper, a Java-based scientometric software package named Citespace is used to reduce research bias and increase the quality of review. Citespace was developed to analyze the scientific literature and generate the visual network based on citation records [34,37].

**Figure 5:**
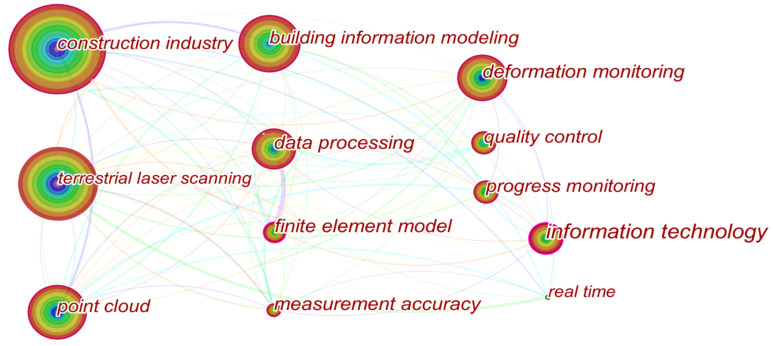
Network of co-occurrence keywords.

(3) Co-occurrence keywords analysis. Keywords are important for indicating the core research contents of papers and capturing the focus and development trend of subject areas over time, and was performed with the help of Citespace. The co-occurrence keywords were extracted from “Title, Abstract, Author Keywords (DE) and Keywords Plus (ID) provided by WOS database” in the software. As shown in Figure 5, each keyword in the network is presented as a node, the size of which is directly proportional to the number of papers containing the established keyword. Meanwhile, the link between different nodes indicates the corresponding keywords appear in a same publication.

The top 10 most frequently occurring keywords are listed in Table 2. To better understand the network, a serious of keywords can be divided into two parts. One is concerned with the preset topic, including “Construction industry”, “Terrestrial laser scanning” and “Point cloud”. The other is about specific applications, such as “Building information modeling”, “Deformation monitoring”, and “Progress monitoring”. In addition, the keywords “Information technology”, “Finite element model”, “Data processing”, “Deformation monitoring”, “Progress monitoring” and “Building information modeling” receive a high value of centrality, and are considered to be pivotal nodes in the network, likely exerting greater influence on others.

**Figure 6:**
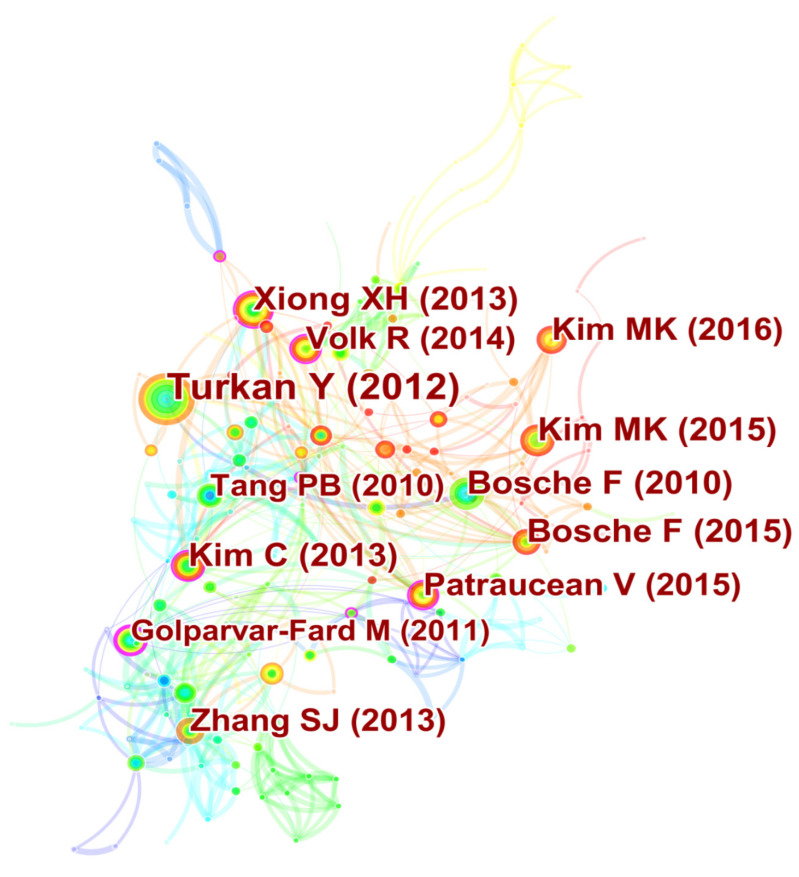
Network of document co-citation.

(4) Document co-citation analysis. Document co-citation network is used to demonstrate the most frequently cited and influential research references, on the basis of which new researchers can easily get involved in a specific research domain [38]. As shown in Figure 6, each node, labeled with the representative author’s name and the year of publication, stands for a cited paper. The top 5 high-frequency cited and high-centrality papers are listed in Table 3 and Table 4. These important papers cover the major applications of TLS in the AEC industry and provide a critical reference for subsequent research. Specifically, they can be divided into five categories, basically including: (a) 3D model reconstruction. Golparvar-Fard M et al. [39], which receives the highest centrality score, compared image-based with laser scanning reconstruction and modeling approaches for as-built project status. Volk R et al. [40] and Patraucean V et al. [41] provided a general overview of BIM creation and implementation from data collection to BIM generation. (b) Progress tracking. Turkan et al. [42], which has been cited the most frequently, developed a progress tracking system by realizing a 4D model (combination of 3D model and schedule data) on the basis of scanning data. Kim C et al. [39] presented a construction progress measurement system consisting of three phases: alignment of the scanning data with the as-planned model, matching of the scanning data to information in the BIM, and revision of the as-built status. (c) Object recognition. Xiong XH et al. [37] presented an automated method for identifying and modeling the main structural components of a building from point clouds. Bosche F et al. [38] improved the algorithm for the recognition of 3D CAD model objects in construction scanning data and proposed an algorithm for automatically calculating the as-built pose of the recognized CAD objects. Bosche F et al. [43] presented a method based on Hough transform to automatically recognize and identify objects with circular cross-sections in scanning data acquired from construction sites, given the project’s 3D design BIM model. Walsh SB et al. [44] outlined the key steps required for processing point clouds and developed data processing algorithms from raw scanning data. (d) Quality assessment. Kim MK et al. [45] developed a holistic framework for the dimensional and surface quality assessment of precast concrete elements based on BIM and TLS. (e) Integrated application. Fekete S et al. [28] employed a laser scanner for geotechnical assessment in tunnel operation. The collected scanning data were used for further analysis, including lining evaluation, quality control, controlled overbreak analysis and surface characterization identification.

## 4. Research Topics Related to TLS in the AEC Industry

**Figure 7:**
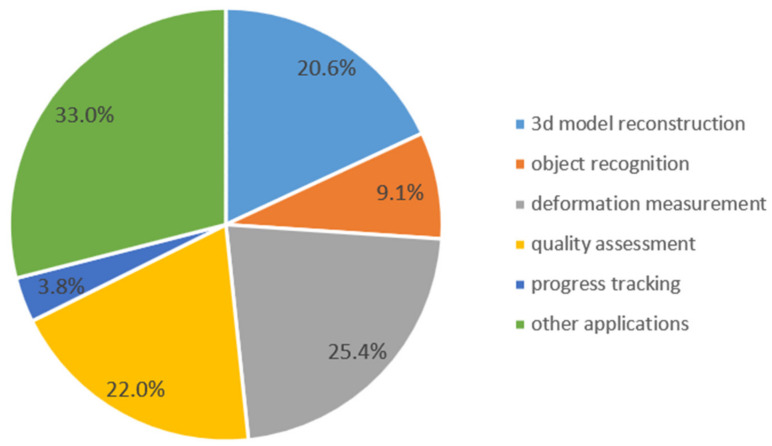
Distribution of applications of TLS in the AEC industry.

The output of the laser scanning process consists of high-density point clouds that contain (x,y,z), RGB, and intensity values, which help to capture the precise geometric data and detailed texture information of the object to be measured. Referring to the network of co-occurrence keywords (Figure 5), an in-depth analysis was performed based on the 209 publications in Set A according to five major applications, including: (1) 3D model reconstruction, (2) object recognition, (3) deformation measurement, (4) quality assessment, and (5) progress tracking. Figure 7 illustrates that the most widely studied topic is deformation measurement, with 53 papers, accounting for 25.4% of all publications. Additionally, another two major applications are quality assessment (22.5%) and 3D model reconstruction (20.6%). Please note that papers published in fields in which papers are seldom published and those without any specific application, such as those focused on data acquisition, data processing, data quality, etc., are categorized into the “other applications” category in Figure 7. For a clear understanding, a summary of the literature regarding different applications is shown in Table 5.

### 4.1. 3D Model Reconstruction (MR)

**Figure 8:**
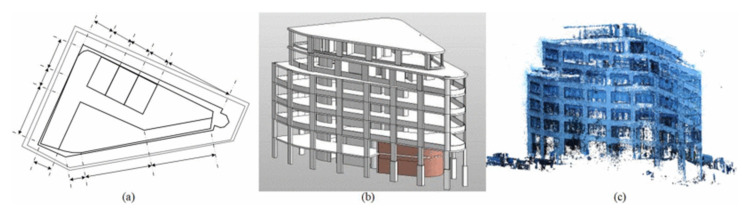
Examples of a building. (**a**) 2D blueprint; (**b**) 3D BIM; (**c**) point cloud [14].

The application of building information modeling (BIM) facilitates information exchange and enhances communication between the various stakeholders over the course of the project life cycle in the AEC industry. Nowadays, BIM is reconstructed with a heavy reliance on 2D CAD drawings, while such a model does not accurately reflect the as-built condition of buildings or facilities (Figure 8). Actually, changes between as-designed information and as-built condition affect the effective use of BIM. In recent years, Scan-to-BIM has become a feasible approach for improving management in the construction and maintenance phases by capturing dynamically updateable as-built information.

Compared to other general techniques like a total station, the main advantage of terrestrial laser scanning is the fast collection of high-density points with x, y, z coordinates and RGB and intensity values, which can be further used in reconstructing a 3D model of objects in construction environments. With the development of TLS and modeling approaches, relevant studies have been conducted on the reconstruction of different objects, including buildings [49,51,53,54,58,60,61,65,70,72], civil infrastructure and its components (bridges [55,62,63,64,73,74,75,76,77], tunnels [50], precast concrete elements [71,79], removable floodwalls [56], bridge piers [66], pipe racks [52]), and construction sites [67,68,69]. The process of 3D model reconstruction using TLS can be classified into three main phases: (1) data collection, (2) data pre-processing, in which the critical task is to register multiple scans in a common coordinate system, and (3) modeling.

Besides BIM, TLS has been developed for use in connection with the finite element model (FEM) and the digital elevation model (DEM). The precise 3D geometric model could be reconstructed from point clouds, which is suitable for the finite element analysis of structural behaviors. Izabela Lubowiecka et al. [82] performed prior research integrating laser scanning, ground penetrating radar (GPR), and finite element analysis (FEM) in historic bridge modeling. The authors applied TLS to obtain the geometry of the structure and used the GPR data to study the internal structure. The resulting information was used to properly define a finite element-based structural model for simulating the structural behaviors of the bridge. Qiu et al. [87] generated a high-resolution DEM of a railway tunnel surface at a resolution of 0.005 m from TLS data for further analysis.

Point cloud data are the basis of 3D model reconstruction, and there is a correlation between modeling accuracy and the number of acquired points of objects [179]. Of all the technologies that can be used to capture point clouds (summarized in Section 4.6), the prominent advantages of TLS in MR application include the measurement accuracy and the amount of data, especially for large-scale and complex objects [39,180,181], while in many applications it captures unnecessarily dense data, wasting time for data collection and processing. When there is a high demand in terms of time or there is no specific need for the modeling accuracy in the project, TLS is not as useful or convenient as other technologies [182]. Thus, the selection of an appropriate method for acquiring point clouds depends on the project requirements and the technical performance of the instrument.

### 4.2. Object Recognition (OR)

In general, three types of knowledge need to be represented in the reconstruction process: object geometric shapes, object identities, and spatial relationships between objects [12]. However, the raw point clouds do not contain any semantic or topological information. To use the massive data for further applications in the AEC industry, it is necessary to process the acquired points to generate a semantically rich BIM. Object recognition aims to detect and classify different types of objects in point clouds by recognizing geometric and semantic information as well as the topological relationships between objects. Strictly speaking, the application of 3D model reconstruction consists of an object recognition process. To make a clear comparison, model reconstruction, as mentioned above, emphasizes the generation of a geometric model, while the identified model of the objects not only contains geometric information, but also object-based semantic information.

Previous researchers have developed different strategies and algorithms for identifying various objects (building components [42,47,70,88,92], bridge components [44,57,75,89,91,95,97], tunnel components [78,98], construction site [96], and construction equipment [68]). The typical approach in object recognition is to use shape descriptors. Using various machine learning methods, the object to be recognized is matched with objects with high descriptor similarity in the model database. Xu et al. [14] and Ma et al. [58] introduced and categorized the most popular approaches to semantic model reconstruction from point cloud data. It is worth noting that with the rapid development of machine learning methods, recognition approaches based on deep learning are attracting increasing attention. Kim et al. [90] compared three deep-learning models, PointNet, PointCNN, and Dynamic Graph Convolutional Neural Network (DGCNN), for classifying the components of bridges.

Nevertheless, recognizing objects from raw point clouds remains a challenging task. Most previous studies involve small objects like structural components. In complex construction site environments, many recognition algorithms fail to maintain a good recognition rate [183]. The reason for this is that it is difficult to obtain complete 3D point data of objects due to confounding factors like noise and occlusion. Furthermore, similarities between different types of objects can also cause problems for classification. Image-based photogrammetry is the most common method for object recognition due to the availability of large amounts of training data. Thus, large point cloud datasets are needed to train classifiers for recognizing objects from point clouds. Hackel et al. [184] provided a good example of solving this problem. Another possible solution is to convert the existing 3D CAD models into point clouds to form the training dataset [183].

### 4.3. Deformation Measurement (DM)

Engineering structures such as high-rise buildings, bridges, tunnels, foundations, etc., are exposed to changing applied loads not only during the construction phase but throughout the entire lifetime of the projects, which generally leads to deformation and structural change. It is of great importance to understand the mechanics of deformation and to check various theoretical hypotheses regarding the behavior of deformed objects, which can be performed mainly through the monitoring and analysis of structures. Over the course of recent decades, the role of deformation measurement has significantly increased. It primarily contributes to providing safety assurances with respect to the monitored object in order to ensure a long lifespan. Meanwhile, deformation monitoring is conducted to provide engineering data for further analysis, such as verifying design parameters, predicting the behavior of a monitored object, and developing measures for implementation in the case of accidents. In comparison with other types of surveys, the higher requirements with respect to the accuracy, periodicity and repeatability of observations are the main distinguishing characteristics of deformation measurement [185]. Considering the above situations, TLS is introduced to monitor the structure, as it is able to provide more complete information, and the chance to extend the capacity of region of deformation monitoring.

Deformation measurement is focused on changes in the relative position of a structure, which requires the collection scanning data at a certain interval to maintain periodic monitoring of the structural response by comparing data at different time points. Various case studies have been carried out on the monitoring of engineering structures, including buildings [104,145], dams [113,122,126,130], bridges [59,100,105,106,108,109,110,116,118,119,121,123,124,128,137,144], tunnels [57,80,103,107,114,115,117,125,127,129,131,132,134,135,136,139,140], stations [99,143], foundation pits [186], pipe racks [52], towers [101], and many others. In addition, another main direction of studies for deformation measurement is to perform structural health monitoring of infrastructures that are in service for a long time, especially for masonry [99,144,187,188,189] and wooden structures [190,191,192].

According to the current standards, the deformation of structures is monitored based on critical points. There is no doubt that the ranging accuracy of TLS is not as high as traditional instruments like levels and total stations [111,193]. In addition, traditional instruments are more suitable for high-frequency deformation measurements carried out on the basis of capturing and processing limited point data [194]. However, the exploitation of scanning data with high redundancy is the key to deformation analysis [195], and the accuracy can increase depending on the analysis techniques. Various approaches have been developed to analyze structural alterations in terms of shape or dimensions, and these can be categorized into four major groups: point-based methods, point-to-surface-based methods, surface-based methods, and geometry-based methods [108]. Additionally, point-based methods represent not only point-to-point but also point-cloud-to-point-cloud approaches. In practice, except for point-to-point-based deformation analysis, which often relies on artificial targets such as spheres and retro-reflective targets placed on a deformable object, the other methods do not require auxiliary targets, and data captured from different epochs can be directly compared to calculate the deformation after transformation into a common coordinate system.

### 4.4. Quality Assessment (QA)

Quality assessment consists of three parts, depending on the different target problems: construction quality management, dimensional quality inspection, and surface quality inspection. For all these applications, the key is to extract geometric and semantic information from point clouds of objects.

Construction quality management concentrates on the process monitoring and control of construction activities with the intention of meeting requirements of the design plans and specifications. In this regard, the majority of research has focused on assembly process management for prefabricated components. Zhou et al. [76] proposed a framework for the virtual trial assembly (VTA) of steel structures with bolted connections to reduce on-site assembly discrepancies. After collecting high-precision point clouds of prefabricated segments, a finite element analysis is performed on the basis of the reconstructed BIM to simulate the deformation and stresses caused by forced assembly. Kim et al. [148] and Jeng et al. [147] used TLS to capture the geometric and position information of prefabricated components in the process of bridge assembly and construction. Xu et al. [131] examined the feasibility of using point cloud data for near-real-time quality inspection of newly assembled circular tunnel shield segment rings. In addition, some papers have applied TLS to calculate the excavated volume in order to evaluate the excavation quality of different tools [146,150,151].

A part of industrialization, prefabrication has become a popular construction component in the ACE industry. Compared to cast-in-place construction, precast elements offer faster production, lower cost, and a cleaner and safer construction environment [79]. However, the use of precast elements can suffer from unexpected delays and unavoidable increases in cost during construction if the compliance of the precast elements with dimensional tolerances is not properly assessed. To avoid failure during on-site construction, efforts should be made towards performing a comparison between the dimensional conformance of precast elements in as-designed status and as-built status. Research efforts have covered a wide range of precast elements, such as concrete elements (walls [56,71], columns [153], stairs [71], slabs [45,79,92], hollow spheres [155], bridge piers [66]), steel structures [47,156], pipes [154,157], and joinery products [152]. Obviously, the key point in dimensional quality inspection is that the fabrication model of precast elements constructed from point cloud data should be compared with the corresponding as-designed BIM model in a common coordinate system in order to identify dimensional discrepancies. Additionally, it makes sense to perform dimensional inspection on infrastructures that have been in service for a long time. The point cloud data obtained by TLS can be used not only to effectively document historical buildings and structures, but also to provide useful geometric parameters for structural analysis. Studies in this area have focused on masonry [188,196,197,198,199,200,201,202,203,204] and wooden infrastructures [191,192,205,206,207,208].

Many existing structures suffer from damage due to age or accumulated damage from hazards [89]. Surface quality inspection mainly refers to the inspection of the present conditions of a concrete surface, which is important for assessing the safety and reliability of a structure. The most researched topics related to surface quality inspection can be grouped into flatness assessment and structural damage identification. In general, there are two common methods for surface flatness inspection that can be found in previous studies: (1) following quantitative indexes defined in relevant standards such as the F-numbers method [163,172]; or (2) setting up a reference plane and calculating the deviations between surface points and the reference plane [162,170]. On the other hand, the majority of papers on structural damage identification are focused on surface cracks [89,98,99,104,144,171,174,175], spalling [75,89], corrosion [161], water leakage [173], and concrete loss [160,166].

### 4.5. Progress Tracking (PT)

The effectiveness of TLS applied in monitoring construction activities progress has been validated in a number of previous studies [42,54,92,177,178]. As with quality assessment, object recognition plays an important role in progress monitoring. Individual as-built components need to be recognized from the point clouds and compared with the corresponding as-designed part to track the progress. Turkan et al. [42] made an important contribution to this research topic. In their article, a 3D CAD model combined with schedule information was used to provide the designed spatial characteristics of the facility under construction over time, and scanning data were used to provide the current site conditions. The proposed system required the point clouds and the 4D model to be registered in the same coordinate system to be able to extract useful data for progress tracking. Furthermore, the critical point was the identification of objects from point cloud data, and the construction progress to date was calculated by the system on the basis of the object recognition results from the analysis of the scans acquired on that date. In recent years, the integration of multiple technologies developed for monitoring construction progress has become popular. Braun et al. [88] presented a method for improving the accuracy of construction progress monitoring by fusing point clouds, semantic data, and computer vision. Their contribution to the combination of methods was the introduction of a CNN-based object-detection method to correctly detect elements that otherwise tend to be falsely classified. Ali et al. [53] proposed a near-real-time construction progress monitoring system called iVR. Specifically, the iVR consists of five modules: iVR-location finder (finding laser scanner located in the construction site), iVR-scan (capturing point cloud data of job-site indoor activity), iVR-prepare (processing and converting 3D scan data into a 3D model), iVR-inspect (conducting immersive visual reality inspection in the construction office), and iVR-feedback (visualizing inspection feedback from the job-site using augmented reality).

### 4.6. Other Applications

#### 4.6.1. Performance Evaluation of Terrestrial Laser Scanners

The reliability of point cloud data is critically important to the application of TLS in the AEC industry. Accordingly, users need to evaluate the performance of instruments to determine whether they meet the technical specifications supplied by the manufacturers or the project requirements. Several studies have made efforts to verify the performance of scanning systems under laboratory or field conditions to determine their consistency with technical specifications [111,112,209,210,211,212,213,214,215,216]. Evaluating the ranging accuracy and point accuracy are critical procedures for performing the test. In general, the test is verified by establishing reference values with a high-accuracy instrument such as a laser tracker [217,218,219] and a total station [112,210,220]. Additionally, system calibration and performance evaluation are closely related topics. By quantifying and correcting the influence of specific systematic errors, periodic system calibration is critical for ensuring the reliability of the data. Some papers have developed self-calibration methods and procedures [221,222,223,224,225,226]. The publication of the ASTM E2938-15, ASTM E3125-17, and ISO 17123-9 standards enables objective comparability between the various instruments. Wang et al. [227] and Shi et al. [228] provided a reference for understanding and implementing the standards.

#### 4.6.2. Comparison of Different Techniques and Tools for Capturing 3D Point Clouds

3D point clouds are currently most commonly acquired by a terrestrial laser scanner. However, several limitations are found in TLS applications, such as the high equipment cost and the restricted access [229]. Several studies have been conducted on different techniques and tools for obtaining detailed point clouds in the AEC industry, including laser scanning, photogrammetry, and videogrammetry, as explained in the following. The comparison of different methods is shown in Table 6.

(1) Laser scanning

Apart from TLS, there are other two types of laser scanning based on different working platforms: airborne laser scanning (ALS) and mobile laser scanning (MLS) [16]. TLS is ground-based and usually mounted on a static tripod. ALS refers to a scanning system mounted in an aircraft such as UAV or helicopter. MLS can be mounted on land-based mobile platforms such as cars or robots. These three systems differ in terms of scanning mechanism, speed, accuracy, etc. Therefore, each system has different advantages and is suitable for certain projects. ALS and MLS can scan large areas quickly and survey areas with limited accessibility, while TLS allows for more detailed point clouds with relatively high precision and low cost [230,231,232,233,234].

(2) Photogrammetry

Photogrammetry works by taking high-resolution photographs of a scene from different locations via cameras and then processing photos through programs to obtain the spatial information of objects. Depending on the number of cameras, Structure from Motion (SfM) and Multi-View Stereo (MVS) are the mainstream methods for image-based 3D reconstruction and point cloud generation. The traditional photogrammetric instruments include single-lens cameras, stereo cameras, RGB-D cameras, etc. Due to the advancement of camera technology and image processing algorithms in recent years, smartphone-based [235,236,237] and UAV-based [229,238,239,240,241,242] acquisition methods of point clouds have been developed and applied. The key benefit of most photogrammetric instruments is real-time acquisition (portable and flexible) at low cost. However, several limitations can be found in the previous studies, among which the most significant are the lower accuracy, especially in large-scale environments, compared to TLS and the less automated process, which leads to more error [39,243,244,245].

(3) Videogrammetry

Videogrammetry is similar to photogrammetry, but extracts point cloud data from video streams. It can be used with a single camera, a stereo camera, or a multi-camera system to collect video frames and then recover the 3D spatial information of the objects. In the case of a single video stream, since the information from each video frame builds upon the previous one, the sequential characteristic of the video frames makes it possible to progressively reconstruct the detailed spatial information [246]. In comparison with photogrammetry, the videogrammetric method requires little human intervention in the data capturing process, and is appropriate for dynamic object reconstruction [246]. However, the feasibility of videogrammetry in the AEC industry suffers significantly from the quality of the captured frames and the computational process [243,247]. It is a major concern that the quality of video frames is poor compared to still images. In addition, considering the complexity of a construction site, a video sequence consists of many frames, making performing post-processing computationally expensive. As a result, only a few studies have used videogrammetry to obtain point cloud data [248,249,250].

#### 4.6.3. Integration of Digital Technologies

Currently, the AEC industry is experiencing rapid digital transformation, and an increasing number of advanced technologies will be developed and introduced in the AEC industry in future. With the rapid development of various digital technologies, the advantages of using a single technology are becoming weak, and they are not able to satisfy the comprehensive management requirements on site. Accordingly, integrated development and the application of multiple technologies are topics that have been brought to the fore. These approaches can compensate for the drawbacks of individual technologies, resulting in improvement in the productivity and accuracy of the collected data. BIM is the most relevant technology for TLS, as summarized in Section 4.1. Additionally, various technologies have been integrated with TLS to achieve better results in application. Apart from the above techniques for capturing point clouds, common techniques include:

(1) Ground Penetrating Radar (GPR) and Infrared thermography (IR)

As non-destructive techniques, GPR and IR provide an effective complement to TLS in structural evaluation [251,252,253,254]. Lafi et al. [255] demonstrated that IR and TLS are the most useful automatic tools for monitoring and assessing civil infrastructure conditions. TLS is able to capture the precise external geometry of structures, while GPR and IR provide valuable information for detecting internal and sub-surface structural elements. For instance, Puente et al. [251] integrated mobile and static light detection and ranging devices to analyze the exterior of a bridge, while ground penetrating radar equipment was used to characterize its internal stonework.

(2) Digital Twin (DT)

DT technology incorporates three key components—the physical entity, the virtual entity, and the connection of data—to form a practical loop [2]. Several papers have discussed the inclusion of TLS in DT [57,256]. Firstly, point cloud data of the physical object are collected and transferred to the virtual environment. Then, solutions are provided to predict and guide the realistic process by processing and optimizing the data in a virtual model. However, research has seldom [256] carried out the second step for further application, with most studies focusing on the first step. Research aiming towards the integration of TLS and DT remains a challenge.

(3) Virtual Reality (VR) and Augmented Reality (AR)

In recent years, due to the advantage of providing an engaging environment, immersive technologies like VR/AR have been tentatively applied to simulate hazardous construction scenarios and to conduct construction engineering education and training. Duer et al. [257] and Shanbari et al. [65] showed TLS and VR/AR to be a useful tool for documenting the existing conditions of buildings for education management.

Additionally, some researchers have tried to use point cloud data for construction safety management [258,259,260,261]. In these papers, computational algorithms were developed to automatically identify spatial blind spots from the collected point cloud data of heavy construction equipment in field environments. Furthermore, Nguyen et al. [67] integrated BIM and TLS to improve the efficiency of the quantity management process.

## 5. Critical Issues in Application

### 5.1. Workflow

**Figure 9:**
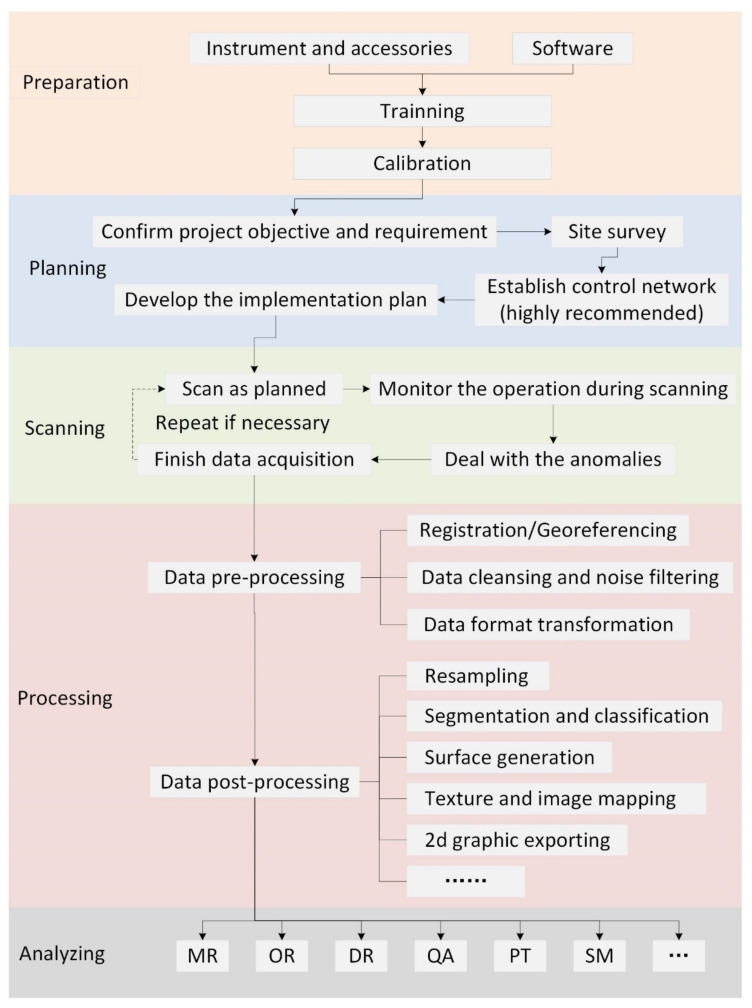
Workflow of TLS.

As numerous studies and applications have been conducted in the AEC industry, it is of great significance to create general rules for TLS-based workflows for the purposes of widespread adoption. Based on the literature review, in this section, an integrated framework is developed, in which the proper use of TLS in the AEC industry is promoted. The framework, shown in Figure 9, provides a detailed workflow for TLS and specific considerations at each step. The framework is composed of five essential stages for performing a typical scanning job, each of which consists of a series of steps. The specifics of the framework are described in the following.

Preparation: As the basis for a scanning work, this stage primarily deals with the preparation of the equipment used to collect field data, including the instrument and accessories, software, and the necessary work to ensure the appropriate use of the instrument. At present, choosing the appropriate scanner is a question of budget as well as theoretical and practical requirements. The features that need to be considered mainly include: accuracy, range, speed, portability, field-of-view, operating environment, etc. Furthermore, the selection of software primarily depends on the deliverable requirements and the capabilities of the software. Regardless of whether purchasing or renting a laser scanning system, it is an essential requirement for users that they should be familiar with the instrument in order to be able to carry out the work properly and effectively. The instruments should be operated only by experienced persons and those who have received training. Since the long-term use of instruments may reduce their precision and resolution, calibration of surveying instruments should be carried out periodically and before application. Standard calibration procedures and proper calibration techniques contribute to minimizing systematic error and ensuring the long-term reliability of the instruments.

Planning: Before conducting field operations, a strong emphasis has to be placed on planning work, as in most engineering projects. The following factors should be considered: (1) confirm project objectives and requirements, including objects to be scanned, accuracy required, time constraints, deliverables, etc. (2) Site survey. Site conditions should be considered to determine the scanning scheme (pre-scan if needed). (3) Establish control network (highly recommended). It is critical to establish a control network for high-accuracy work, especially in the AEC industry. This delivers the basis that links the local coordinate system to a global coordinate system. (4) Develop an implementation plan. The plan scheme contains at least the following important points: selection of optimum scan position, specification of scan parameters, and determination of the schedule of data collection.

Scanning: Start the field work according to the plan scheme. The target object should be scanned in as much detail as possible in the first survey. If the site conditions do not correspond to the plan, the operator should select more suitable locations and parameters. Additionally, monitoring the operation at each scan position is an important step in the scanning process. The operator should note if and when the system encounters difficulty and should be prepared to take appropriate action to ensure data quality. Anomalies during scanning should be documented and dealt with in a timely fashion. Such unexpected situations can include: unfavorable weather conditions, disruptions, problems, accidents, etc. When all the planning works are completed, it should be determined if have been achieved preliminary objectives of data collection. It is highly recommended that the accuracy of scan registration in the field be checked whenever possible to ensure data quality.

Processing and analyzing: The processing of the raw scanning data is essential prior to the further analysis. Details are described in Section 5.4. Depending on the objectives and deliverables of the project, the processed data will be evaluated using a variety of different techniques and methods. In practice, the analysis procedure and algorithms make a great deal of difference in specific applications, as summarized in Section 4.

### 5.2. Data Quality

**Figure 10:**
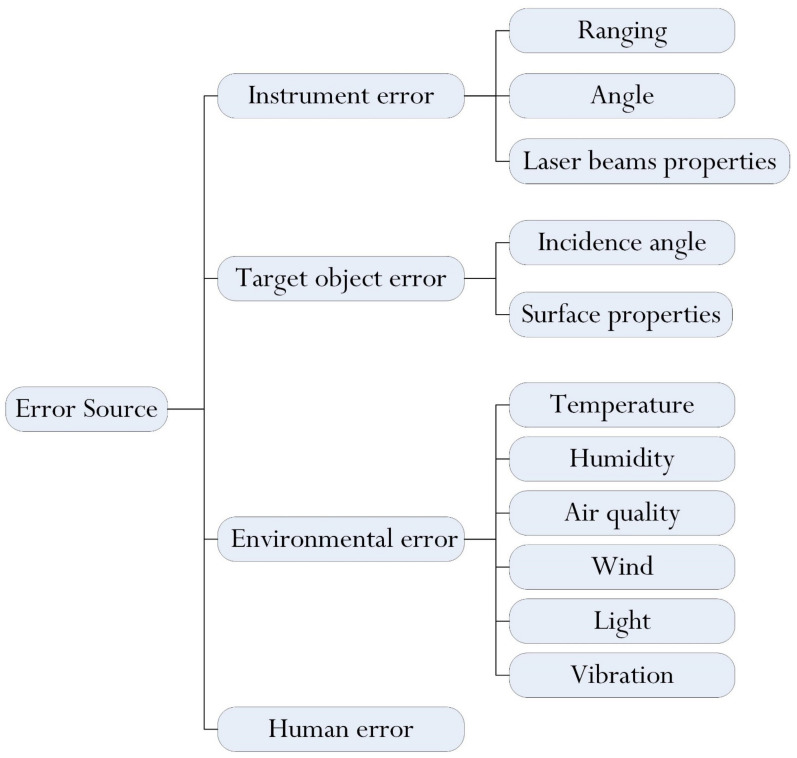
The classification of error sources.

Accuracy in the millimeter range or higher is a typical standard for high-precision applications in the AEC industry, such as structural assessment and assembly management. As a result, data quality is one of the most important factors in whether TLS can be used effectively in the AEC industry. As in the case of conventional technologies, TLS is also subject to different sources of uncertainties during the surveying process. In this case, it is critical to identify the underlying sourceds of error that influence data quality and to evaluate their effect on the results. As shown in Figure 10, a list of error sources is summarized and categorized by cause. In general, uncertainties can be grouped into four broad categories in terms of whether they are related to the instrument, the target object, the environment, or the operator. (1) Instrument error may be further partitioned into ranging error, angular error, and beam property error. (2) Target object error is associated with the incidence angle of the laser beam on the object surface and surface physical properties. (3) Environment error is largely related to atmospheric and environmental effects on the scanning device. (4) Human error arises from the process of operating instruments depending on the skill and experience of operators.

A considerable number of studies have addressed the error analysis and performance evaluation of laser scanners, which is critical for ensuring adequate data quality and reliability. JavierRoca-Pardiñas et al. [262] proposed an error model for TLS measurements, in which the error was estimated on the basis of the distance to the object and the angle of incidence. Wang et al. [263] introduced a combined model by integrating external models related to atmospheric refraction, beam wander and incidence angle into a seven-parameter similarity transformation model to detect external errors and register multiple scans. Kerekes et al. [264] presented a stochastic model for TLS observations. By classifying the atmospheric parameters as stochastic correlating elementary errors, the currently elementary error model is expanded. Ling et al. [211] studied the influence of distance, incident angle, and target color on the accuracy of the scanner. Bolkas et al. [265] demonstrated that users should consider instrument specification, required precision of plane residuals, required point spacing, target color, and target sheen when selecting scanning locations. It should be noted that standardized tests for quantifying the effect of various error sources are still lacking [266]. Thus, demonstrating data quality and reliability can still pose challenges.

### 5.3. Scan Planning

The high efficiency of data acquisition plays an important role in complex and constantly changing construction environments. Therefore, it is necessary to determine appropriate scan positions and parameters so that the quality requirements of the collected data could be satisfied with minimal operating time before the process of data acquisition. The current practice of scan planning relies on the experience of trained operators, for whom conducting a one-off successful scan task with the required data quality is also a challenge. Occasionally, redundant scans are needed to reduce the risk of incomplete and low-quality data, which is time-consuming and inefficient. Accordingly, some researchers have focused on automated strategies for generating optimal scan plans and parameters. Argüelles-Fraga et al. [267] carried out one of the early studies regarding tunnel scan planning. A method was proposed for optimizing tunnel scanning tasks by estimating the angular interval and the maximum scan distance. Cabo et al. [268] described a method for scan planning in tunnels that determined the optimal scanner positions throughout the tunnel. The proposed approach achieved this by finding the largest possible distance between adjacent scanner positions, while satisfying some restrictions with respect to the point density, incidence angle, scanning distance, and placement of the scanner.

**Figure 11:**
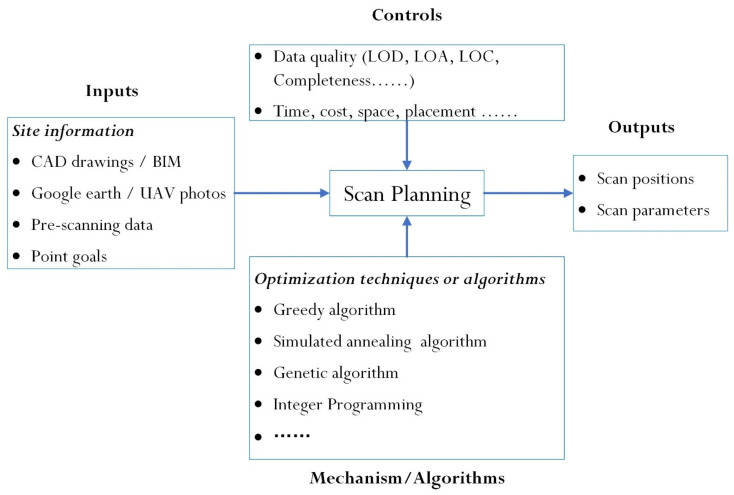
The functional model of scan planning.

A functional model was refined from the previous studies based on the IDEF0 method shown in Figure 11. Scan planning was formulated as a series of optimization problems, with three main elements to the optimization problems: inputs, controls, and optimization models. Aryan et al. [269] reviewed prior publications based on these three problem elements and proposed three main data quality considerations: completeness, accuracy, and registrability.

### 5.4. Data Processing

Data processing is as important as data acquisition in the application of TLS. The successful use of TLS depends not only on the technical specifications of the scanning instruments themselves, but also on the capabilities of data processing to address the data and perform necessary analysis after data acquisition. Before processing point clouds, the data acquired from different positions need to be transformed into a given coordinate system to constitute an entire object. In addition, data cleansing and the filtering of noise from the raw data are necessary to optimize data quality for analysis and to decrease computational load. In some cases, the raw scanning data should be converted into another format, depending on the requirements of the post-processing software.

Many studies have been conducted on processing techniques and algorithms in recent years. In fact, the specific procedure and approaches used for data processing depend on the intended application of TLS, as different objectives may require different approaches to performance evaluation and have different deliverables. Tang et al. [12] and Xu et al. [14] provided a thorough review of the processing techniques and methods for model reconstruction and object recognition using point clouds. Mukupa et al. [13] presented a detailed investigation of robust processing methods for detecting change and deformation, and proposed a three-stage process model for deformation analysis. Kim et al. [45] established an end-to-end framework consisting of a quality assessment procedure for the dimensional and surface quality assessment of precast concrete elements based on BIM and 3D laser scanning.

Dedicated processing software has a considerable influence on the acceptance of TLS. Nowadays, there is a wide range of software tools with different algorithms and application patterns. Some of those software tools have been developed by laser scanner manufactures to be used mainly with manufacturer-specific scanners and are associated with a particular type of data format, such as Faro Scene from FARO. There may be limitations with respect to the processing of point cloud data from these specific scanners when using other software. In addition, there are many packages like PolyWorks from InnovMetric that offer full support for entire data processing workflows, and which are capable of supporting different scanners. Moreover, some software products have been developed for specific functions or applications, the analysis features and algorithms of which differ from package to package.

## 6. Future Research Directions

To facilitate TLS development and application in the AEC industry, the following future research directions are suggested:

(1) Cost control of hardware and software

As mentioned above, the development of TLS in the AEC industry is restricted by both hardware and software. As one of the major barriers, the cost of hardware and software is high in applications at present compared with other techniques, especially for minor projects or those that do not require accurate data. For example, Bhatla et al. [180] and Gautier et al. [270] used handheld digital cameras and depth cameras instead of TLS to generate as-built 3D point clouds due to their lower cost. However, the drawback of these techniques is the insufficient accuracy of their measurements. As a consequence, it is vital to control the cost and maintain a balance between performance and cost for the healthy development of TLS.

(2) Improvement of data processing capability

Despite plenty of studies having been conducted on data processing, considerable gaps still exist between the state of the art and the demands of their application. There is great potential to improve the processing efficiency, effectiveness, and automation level of the algorithms. In addition, the developed algorithms and methods should be application-oriented and universal in highly specialized fields like the AEC industry. Finally, a standard system for the evaluation of algorithm performance should be established in order to select appropriate methods for different applications.

(3) Automatic scan planning

Most of the existing publications solved medium-scale and generally simple problems [269], while working in real construction environments is difficult, owing to the more complicated set of limitations. Future research should be conducted to optimize the main elements in a functional model of scan planning, including: (a) exploring various kinds of input model matching the real environment well; (b) investigating the required data quality for specific applications and establishing the relationship between the required data quality and the scan parameters; (c) developing more optimal solutions for solving constrained nonlinear optimization problems; (d) using AI technologies and optimization algorithms to increase the level of automation and real-time adjustment to reduce manual intervention in data acquisition.

(4) Integration of digital technologies

On one hand, various techniques for acquiring point cloud data compensate for the drawbacks of TLS in terms of cost, convenience, accessibility, etc. On the other hand, the integration of other types of technologies effectively extends the application area of TLS. However, there are still many challenges, such as the fusion of multi-source information, especially point cloud data. In addition, it is difficult to obtain real-time information for most data acquisition technologies. Thus, there is a lack of applications in dynamic scenarios such as safety management. Integration with wireless location and communication technologies is one of the possible directions.

(5) Adoption of artificial intelligence (AI)

As a branch of computer science, artificial intelligence has created tremendous value by revolutionizing the AEC industry. Due to the great advantage of transforming big data into useful knowledge, there is no doubt that AI in collaboration with TLS will be one of the primary future trends in the field of AEC. In recent years, various AI techniques, deep learning in particular, have been found in previous studies to have huge potential in object detection and quality assessment [90,271,272,273,274]. Potential AI-based solutions in future may include: (a) prediction of project activities (e.g., safety, progress, and productivity); and (b) decision-making optimization (e.g., project planning, scan planning, and resource management). In this context, there is an urgent demand for generating training datasets of point clouds for construction activities.

## 7. Conclusions

The appearance of TLS in the field of AEC was relatively recent, but it is attracting increasing interest. This research evaluates the application of TLS in the AEC industry on the basis of scientometric and qualitative analysis. A five-step literature retrieval was conducted to collect relevant papers. It was found that the number of publications increases from 2017, meaning that the topic has been receiving increasing attention in recent years. To reduce research bias and increase the quality of the review, Citespace was used to investigate keyword co-occurrence and co-citation to provide a reference for further analysis.

The five major applications of TLS in the AEC industry were determined, which include 3D model reconstruction, object recognition, deformation measurement, quality assessment, and progress tracking. To promote the widespread adoption of TLS in the AEC industry, on the basis of the discussion of a set of critical issues in application, a general framework of TLS-based workflow was developed. Meanwhile, the sources of error that influence data quality were summarized, and a functional model for scan planning was developed. This study finally indicates the following future research directions in the hope of providing recommendations and direction to researchers: (1) cost control of hardware and software, (2) improvement of data processing capability, (3) automatic scan planning, (4) integration of digital technologies, (5) adoption of artificial intelligence.

In summary, this paper provides a foundation for the widespread adoption of TLS in the field of AEC, making several contributions, as follows: (1) the evolution and status of the use of TLS in the AEC industry is revealed, helping to understand the research topic; (2) the critical issues in application are explored in order to promote effective use in practice; (3) future directions of the research topic are described in order to provide a reference for further research.

## Figures and Tables

**Table 1:** Comparison of different type laser scanners (specifications offered by manufacturers).

Distance Measuring Methods	Typical Products	Maximum Range (m)	Scan Speed (pts/s)	Ranging Accuracy
Time-of-flight	Riegl VZ-400i	800	500,000	5 mm @ 100 m
	Topcon GLS-2000	500	120,000	3.5 mm @ 150 m
Phase-based	Faro FocusS 150	150	976,000	1 mm @ 25 m
	Z+F IMAGER 5016	365	1,100,000	1.6 mm @ 100 m

**Table 2:** List of Top 10 keywords and related network data.

Keywords	Count	Centrality	Year
Construction industry	334	0.13	2010
Terrestrial laser scanning	155	0.08	2009
Building information modeling	144	0.17	2012
Point cloud	117	0.12	2012
Data processing	87	0.20	2012
Deformation monitoring	87	0.18	2011
Information technology	64	0.37	2012
Progress monitoring	40	0.17	2013
Finite element model	33	0.23	2014
Quality control	32	0.14	2012

**Table 3:** List of Top 5 high-frequency cited papers and related network data.

Cited References	Count	Centrality	Year
Turkan Y [42]	57	0.06	2012
Xiong XH [46]	37	0.16	2013
Bosche F [47]	37	0.07	2010
Kim C [48]	36	0.11	2013
Kim MK [45]	35	0.08	2015
Bosche F [43]	35	0.08	2015

**Table 4:** List of Top 5 high-centrality papers and related network data.

Cited References	Centrality	Count	Year
Golparvar-Fard M [39]	0.27	25	2011
Xiong XH [46]	0.16	37	2013
Volk R [40]	0.13	33	2014
Patraucean V [41]	0.13	32	2015
Fekete S [28]	0.12	11	2010

**Table 5:** Summary of applications of TLS in the AEC industry.

Applications		References
3D model reconstruction (MR)	BIM	[49,50,51,52,53,54,55,56,57,58,59,60,61,62,63,64,65,66,67,68,69,70,71,72,73,74,75,76,77,78,79]
	FEM	[74,80,81,82,83,84,85,86]
	DEM	[87]
object recognition (OR)		[42,44,47,57,68,70,75,78,88,89,90,91,92,93,94,95,96,97,98]
deformation measurement (DM)		[52,59,80,84,85,99,100,101,102,103,104,105,106,107,108,109,110,111,112,113,114,115,116,117,118,119,120,121,122,123,124,125,126,127,128,129,130,131,132,133,134,135,136,137,138,139,140,141,142,143,144,145]
quality assessment (QA)	construction quality Management	[74,131,146,147,148,149,150,151]
	dimensional quality inspection	[45,47,56,64,66,71,79,92,152,153,154,155,156,157]
	surface quality inspection	[44,45,64,71,75,89,98,99,104,158,159,160,161,162,163,164,165,166,167,168,169,170,171,172,173,174,175]
progress tracking (PT)		[42,53,54,88,92,176,177,178]

**Table 6:** Comparison of different point clouds acquisition methods.

Technology	Tools	Range (In General)	Accuracy (In General)	Cost (In General)
Laser scanning	TLS	Moderate	0.5–10 mm	High
	ALS	Long	>10 mm	High
	MLS	Moderate	>10 mm	High
Photogrammetry	Smartphone-based	Close	>10 mm	Low
	UAV-based	Moderate	>10 mm	Moderate
Videogrammetry	Smartphone-based	Close	>10 mm	Low

## Data Availability

The data presented in this study are available on request from the corresponding author.
